# Supplementary material for: Chromothripsis during telomere crisis is independent of NHEJ, and consistent with a replicative origin
Source: Genome Res. 2019 May;29(5):737–49. doi: 10.1101/gr.240705.118 (PMC6499312; doi:10.1101/gr.240705.118)
Supplement: Supplemental Material [file supp_gr.240705.118_Supplemental_file_1.zip › contigs/annotated_contigs/DB113/contig.2.DB113_length_603_mean_cov_8.2056384743.docx]

**DB113_length_603_mean_cov_8.2056384743**

```
AATGCCCAAAACAGTTTGTGTGCCAGTATATATGTGCATGAATTTCTCTATGATTGGCAGTTAAGTGGAAAAGCGGGGTTATAAGACAT
 >chr5:2795441-2795752 + E=2e-175
GGCTTACGATCTCTACTAATACCATCTGAATCTTGTGGTGATGGTGATGATGATGATGAAGACACTGGTATTCGACTACTGAGTCAATT

TTTAAAACTGTGTAGATTTATTTATATGTGTTATTTCTTCTGGCCTCTGTTTTAGAAATAAATATTAATCTTGTAATTTGACCATTTCA

TCTAAGTTTTCAAATCTGGTTTAGGAAACCACTTAGTCTAACCT|TAGTCTAA|CCGCCTTCATAGCTGTCTTGGCGACAAGCTGTGGG
                                                     >chr5:2796814-2797098 + E=3e-159
TCAGGATTTAGGATGGTGTCTACCGGACTCTTAGGTACCTTCCTCCGAGAGAGCCCCATTTGTAATCTATTTTACCCTTCAGTGTAGTA

TTACGTGCCTGCAAAAGTGATATTTCTGAGGATTGATACTCCAATACATCATAATCTGAAGGTTTTTAGGTTTATACTTTGTGTTCTGA

ACTAAGCAGATAACAAGAGCTCACTTAGTCATTTCTCCGATAAAAAGTTATCTTCAAAGAAGAGGACAATT
```
